# Compliance of cigarette packaging with COTPA: Observations from a sub-district setting in southern India

**DOI:** 10.18332/tpc/223966

**Published:** 2026-10-14

**Authors:** Malavika Sachith, Kibballi Madhukeshwar Akshaya, Makthal Keertana, Kaiser Khadija

**Affiliations:** 1 Department of Community MedicineYenepoya Medical CollegeMangaloreDakshina Kannada, KarnatakaIndia

**Keywords:** cigarette smoking, tobacco smoking, health policy, tobacco industry, COTPA

**Dear Editor,**

Tobacco use is a major global public health problem and remains a leading cause of preventable morbidity and mortality^1^. The Cigarettes and Other Tobacco Products Act (COTPA), enacted in India in 2003, incorporates several evidence-based regulatory measures to reduce tobacco consumption and protect public health. Sections 7–9 of COTPA mandate specified health warnings on tobacco product packages, which is a cost-effective strategy intended to inform users of health risks and discourage use^2,3^. We aimed to assess compliance with the implementation and enforcement of Sections 7–9 of the COTPA regarding cigarette packaging in a sub-district of Mangalore in coastal Karnataka, India.

The size, clarity, legibility, conspicuousness, and language of health warnings on cigarette packets were assessed in November 2025. Five operationally defined neighborhoods (a municipal area, a village panchayat, areas near an educational institution, a bus stand, and a recreational site – beach) in a sub-district of Mangalore, coastal Karnataka, were purposively selected to represent diverse retail settings. Tobacco outlets were identified using a standardized walking protocol^4^. At each outlet, empty packets of all unique cigarette brands were collected. Compliance with Sections 7, 8 and 9 of COTPA was independently evaluated by two researchers, with discrepancies resolved in consultation with a third researcher.

A total of 36 unique packets (representing 12 brands) from 45 retail outlets surveyed were included in the analysis. As per the guidelines, pictorial health warnings are mandated to appear on cigarette packets and must change annually. So, 10 out of 36 (27%) packets did not comply with providing the message ‘Tobacco causes painful death’, and they also lacked the mandatory Quitline number (Figure 1A and B). These non-compliant packets also failed to follow the prescribed color scheme, lacked pictorial warnings, and, in some cases, displayed warnings only on side panels, in a very small font, or in a language different from that used to specify the brand name. All such packets were foreign brands marketed in India. Notably, one brand had compliant India-specific packaging, while its imported version was non-compliant. Misleading descriptors like ‘light’, ‘ultra light’, etc, were present on 24 (69%) packets, often placed inside the opening flaps, and 28 (78%) packets featured symbols, colors, or brand elements suggesting reduced harm (Figure 1C).

**Figure 1:**
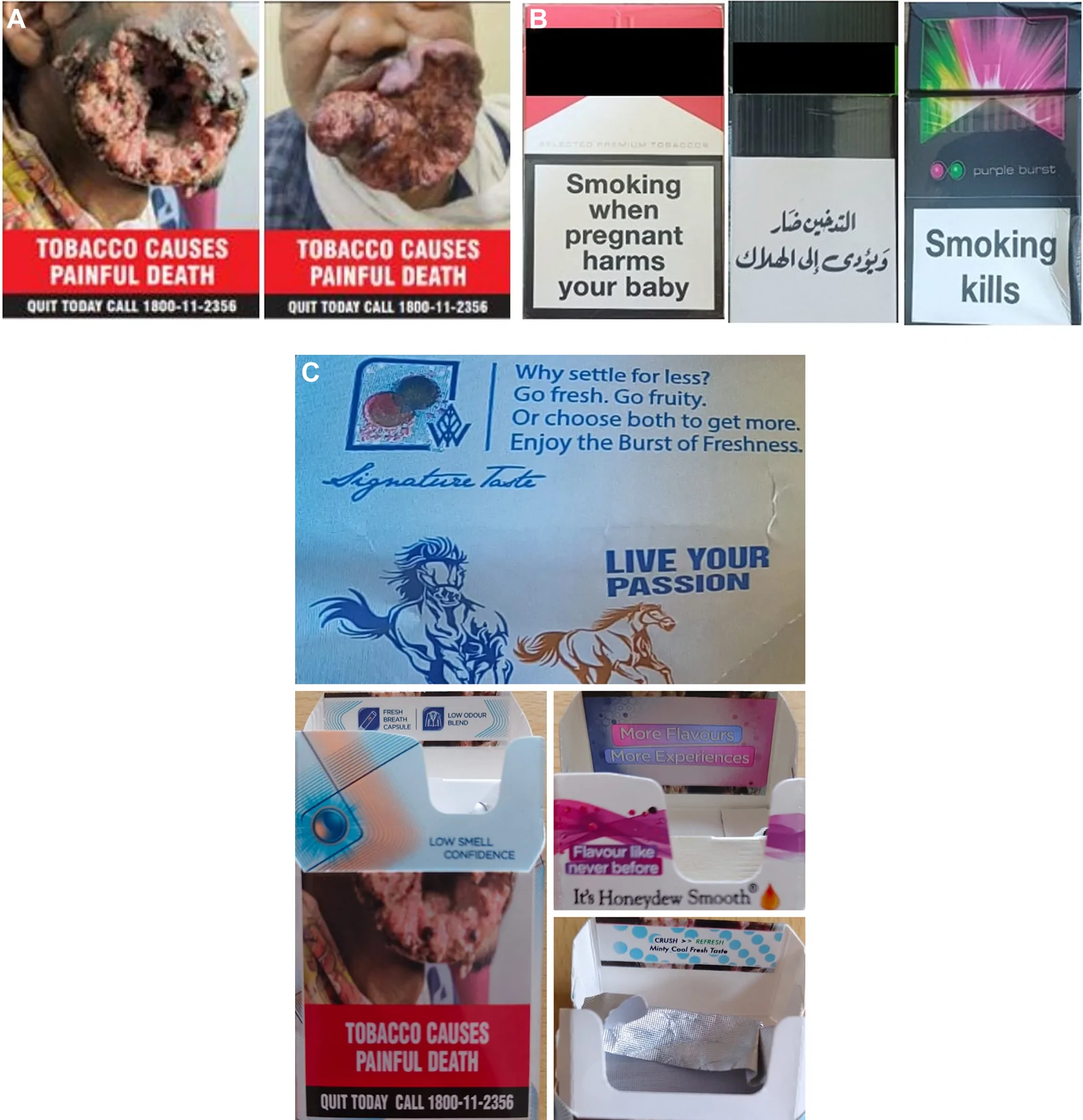
Examples of cigarette packets observed in retail outlets of coastal Karnataka, India, November 2025: A) compliant; B) non-compliant; and C) with misleading descriptors, as per the COTPA Sections 7–9

The evolution of pictorial health warnings in India has been gradual and contested, reflecting persistent tobacco industry resistance^5^. As per COTPA, 85% of the front and back of cigarette packets must incorporate pictorial health warnings. Though, India adheres to these requirements, it stands at ninth position in global comparison as per the cigarette package health warnings international status report published by the Canadian cancer society^6^. In contrast, Nepal achieved 100% coverage in 2025, with evidence showing that 90% coverage prompted less than half of surveyed users to attempt quitting and deterred initiation among nearly one-third of respondents, suggesting that stronger implementation in India could substantially reduce smoking prevalence^7,8^. However, our observations reveal compliance gaps; only 22% of packets met COTPA requirements, echoing findings from a neighboring district and indicating widespread weak enforcement^9^. Foreign brands were uniformly non-compliant, consistent with evidence from Delhi, highlighting the need for stricter monitoring, penalties, and investigation into the entry of non-compliant foreign products into the Indian market^10^.

In summary, our findings highlight the potential weak enforcement of Sections 7–9 of COTPA, despite strong legislation, and emphasize the need for strengthened regulatory monitoring and enforcement to ensure effective tobacco control.
